# Amphiphilic Copolymers and Their Role in the Study of Membrane Proteins

**DOI:** 10.1021/acs.jpclett.5c00680

**Published:** 2025-06-03

**Authors:** Gestél C. Kuyler, Elaine Barnard, Randy D. Cunningham, Sinothando Sibariboyi, Luke White, Ilanie Wessels, Michael-Phillip Smith, Bennie Motloung, Bert Klumperman

**Affiliations:** † Department of Chemistry and Polymer Science, 26697 Stellenbosch University, Private Bag X1, Matieland 7602, South Africa; ‡ Centre for Health and Life Sciences, Coventry University, Coventry CV1 2DS, United Kingdom

## Abstract

Amphiphilic copolymers have revolutionized the study of membrane proteins (MPs), where MPs are known to be critical targets in pharmaceutical development due to their roles in various physiological processes. Traditionally, MP extraction has relied on detergents, which often compromise the protein integrity. Advancements over the past 15 years include the use of poly­(styrene-*co*-maleic acid) (SMA) to form nanoscale SMALPs (styrene maleic acid lipid particles), enabling detergent-free MP extraction. SMALPs preserve the native environment of MPs, facilitating their detailed analysis through a wide range of biophysical techniques. Despite their advantages, SMA-based technologies face challenges such as sensitivity to divalent cations and instability under low pH conditions. Ongoing research focuses on developing next-generation polymers with enhanced properties, utilizing controlled polymerization techniques to obtain narrow molecular weight distributions and chain-end functionality. This paper explores various SMA derivatives and alternative polymer systems like poly­(diisobutylene-*alt*-maleic acid) (DIBMA) and polymethacrylates, offering potential solutions to current limitations and expanding the toolkit for MP research and application.

## Graphical abstract

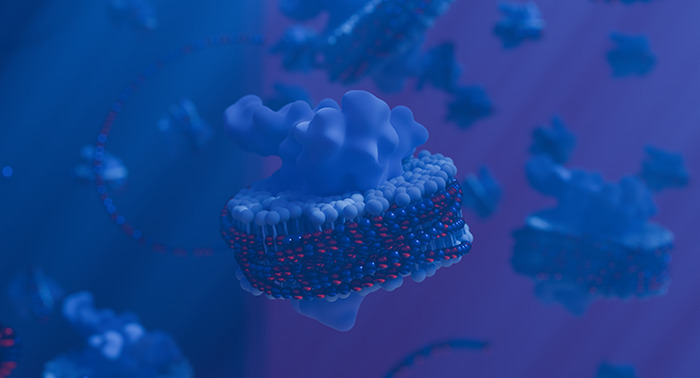

Amphiphilic polymers have become indispensable in membrane protein (MP) research. MPs are key targets in pharmaceutical development due to their roles in various intra- and extracellular functions and physiological processes, such as signaling/communication, transport, adhesion, etc.^1^ In fact, approximately 70% of drugs approved by the Food and Drugs Administration (FDA) in the United States target these important proteins.^2^ Yet, studying MPs presents complexities compared to soluble proteins, as optimal MP function relies on a specialized hydrated lipid bilayer environment.^3^ Preserving their structural and functional integrity during isolation and subsequent analyses is essential for an accurate therapeutic design. Traditionally, MP extraction primarily relies on the use of detergents (i.e., surfactants), which often pose challenges in maintaining the native conformation of the protein and providing a suitable environment for downstream analyses.^4^ Due to the chemical structure of detergents, they can interact with MPs, forming a protective micellar structure that ensures MP water solubility. However, the native conformation of the target protein may or may not be preserved depending on the inherent stability of the protein, detergent type, and biophysical method employed for analysis. Over the past 15 years, amphiphilic polymers have revolutionized MP extraction by disrupting the cell membrane in the complete absence of detergents.^5,6^ This approach stabilizes MPs and their annular lipids in nanoscale discs, facilitating their analysis in a native-like environment while maintaining their physiological properties.

One notable advancement is the use of poly­(styrene-*co*-maleic acid) (SMA) to produce polymer-stabilized nanoparticles (Figure 1(E)), known as SMALPs (styrene maleic acid lipid particles).^6^ SMALPs result from the spontaneous formation of uniform discoidal particles upon introduction of an aqueous SMA solution to biological or synthetic lipid membranes. Unlike conventional head-and-tail detergents, SMA does not form micelles around MPs.^7^ Instead, the polymer is introduced solely during the membrane solubilization step and is not required in buffers used during subsequent purification steps. This simplifies downstream workflows and avoids complications associated with detergent micelles, particularly in light-based detection methods. Polymer-stabilized nanodiscs have enabled compatibility with various spectroscopic and biophysical techniques, including cryo-electron microscopy (EM) and light scattering methods.^8^ Cryo-EM is particularly useful for structural elucidation as it eliminates the prerequisite of challenging MP crystallization, is suitable for a wide range of protein complexes, and is compatible with lipid membrane-mimetic tools.^9^ Thus, cryo-EM allows the resolution of the target protein with its associated lipids, which is particularly important for proteins reliant on lipids for structural and regulatory purposes.^10^

**Figure 1:**
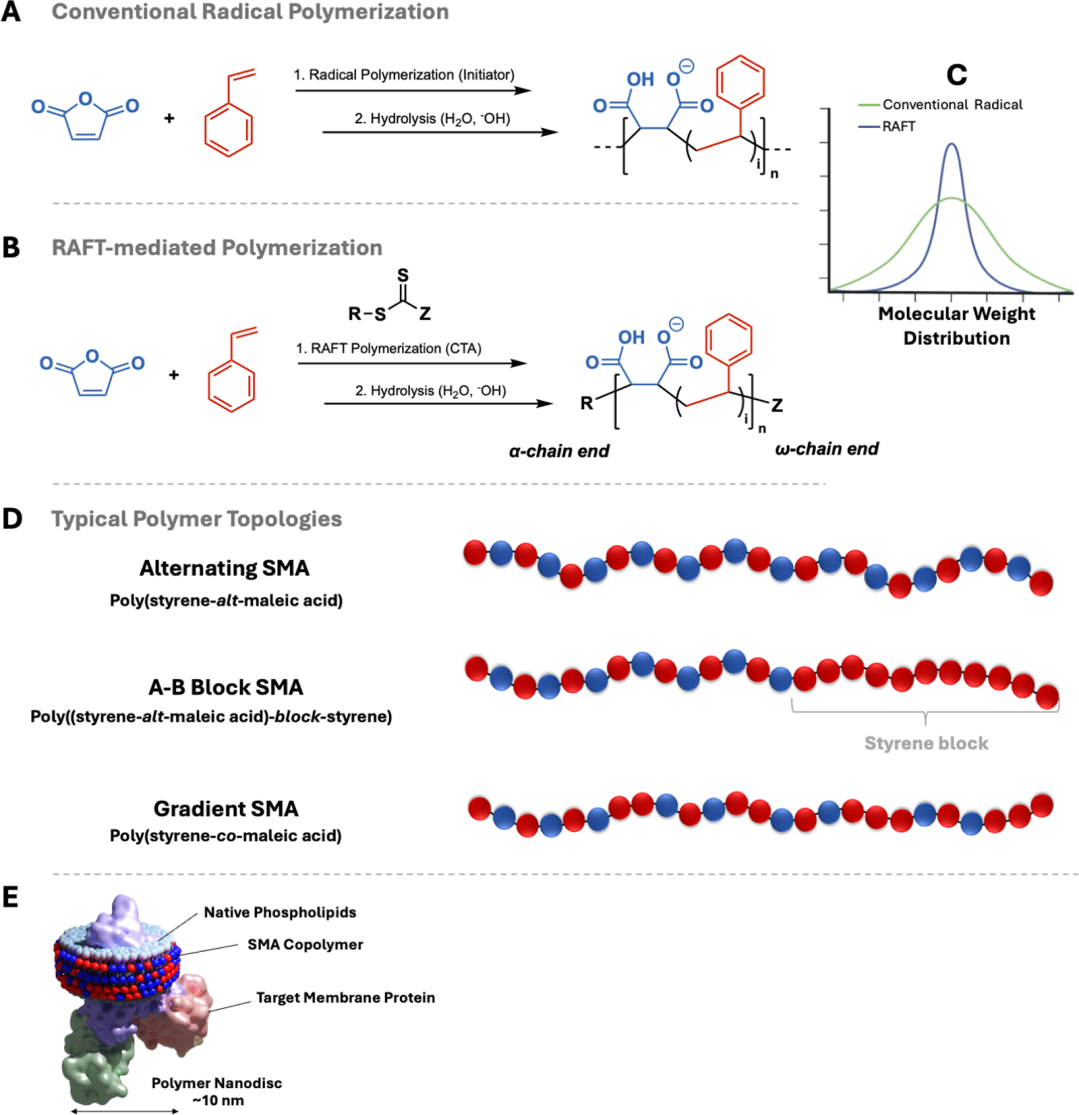
Reaction scheme for the synthesis of SMA via (A) conventional radical polymerization and (B) RAFT-mediated polymerization using a suitable chain transfer agent (CTA). (C) Molecular weight distributions (log M) to illustrate the concept of dispersity in conventional radical vs RAFT-mediated polymerization. (D) Polymer topologies such as alternating, block, and gradient block copolymers are mentioned throughout this section. The arrangement of Sty and MAnh units in these architectures can significantly influence their behavior in membrane solubilization. (E) Graphical representation of a membrane protein (purple/pink/green) in a lipid (light blue) nanodisc stabilized by SMA (red and blue).

While SMA-based technologies have significantly advanced MP research, challenges such as sensitivity to divalent cations, instability at low pH, and restrictions in certain spectroscopic analyses persist.^11^ Ongoing research focuses on the design and synthesis of next-generation or alternative amphiphilic polymers with enhanced properties for the solubilization and characterization of MPs. Furthermore, the implementation of controlled polymerization techniques has been beneficial in methodological and fundamental research,^12–15^ presenting opportunities for further development.

**Poly­(styrene-*co*-maleic acid) (SMA).** SMA is a copolymer comprising hydrophobic styrene and hydrophilic maleic acid repeat units. It is commercially available with multiple variations in polymer characteristics, specifically differing in monomer ratios and molecular weights. Comparative studies demonstrate that the ability of SMA copolymers to solubilize membranes and form nanodiscs is significantly influenced by the polymer chain composition, specifically the styrene to maleic acid (Sty: MA) ratio, with the molecular weight having a less significant impact. The most effective SMA copolymers for membrane solubilization typically have Sty:MA ratios of 2:1 or 3:1, with relatively low weight-average molecular weights of 3 kDa to 10 kDa.^16–18^ Among these, the conventional commercial SMA (2:1) variant remains the preferred choice for MP research due to its solubilization efficiency, high purification yield, and ability to preserve protein function. However, even within a single batch of SMA copolymer, significant variations in composition and molecular weight exist as a result of the synthesis method employed. The unhydrolyzed parent copolymer of SMA, poly­(styrene-*co*-maleic anhydride) (SMAnh), is typically produced via conventional radical copolymerization of styrene and maleic anhydride (MAnh).

Conventional radical polymerization (Figure 1(A)) is widely used for the commercial production of polymers such as SMAnh due to its simplicity and broad applicability.^19^ However, it offers limited control over the molecular weight distribution (MWD), copolymer composition, and polymer architecture because of the probabilistic nature of chain growth. In this method, polymer chains grow through three main stages: initiation, propagation, and termination. The process is initiated by the decomposition of an initiator, generating radicals that react with monomers to form the propagating radicals. Chain propagation proceeds through the sequential addition of monomer units to the propagating radicals. Throughout this process, propagating radicals can undergo chain transfer reactions or termination through a combination or disproportionation. Chain initiation and termination occur continuously throughout the polymerization process, thus, forming new polymer chains at random intervals. This leads to polymer chains of varying lengths and, consequently, a broad MWD (Figure 1(C)). The extent of this distribution is quantified by dispersity (*Đ*). The typical dispersity of conventional SMA ranges from 2.0 to 2.5, indicating highly polydisperse polymers.^20^ For more detailed insights into conventional radical polymerization concepts, readers are referred to comprehensive books by Moad et al. and Matyjaszewski et al.^21,22^

As mentioned, an important polymer property that affects membrane solubilization efficiency is the polymer chain composition, specifically the Sty to MA ratio. This ratio can be altered by adjusting the monomer feed ratio; however, the individual monomers’ chemical properties and reactivities are vital in influencing the polymerization mechanism and final attainable polymer composition. MAnh, an electron-poor monomer, is unlikely to undergo homopolymerization (homopropagation) and thus unable to form MAnh–MAnh diads within a growing chain. Therefore, MAnh reacts almost exclusively with Sty. In contrast, Sty, an electron-rich monomer, easily homopropagates. Yet, when combined, these two monomers create a system in which the homopropagation rate constant for each monomer is low. Conversely, the rate constant for cross-propagation is high, forming SMAnh copolymers with a highly alternating structure (Figure 1(D)) when a 1:1 molar ratio of Sty to MAnh is used.^23,24^

Another factor affecting the monomer sequence of the resultant copolymer is the synthetic conditions used, such as batch-wise polymerization, compared to the continuous stirred tank reactor (CSTR) method.^25^ In research settings, SMAnh synthesis typically occurs through batch polymerization. Nonequimolar SMAnh (Sty:MAnh molar ratio >1) produced via conventional radical polymerization in a batch process is generally a very heterogeneous product, exhibiting broad chemical composition distributions (CCDs) alongside broad MWDs.^24,26^ The underlying phenomenon, known as composition drift, occurs when one monomer is preferentially consumed during the reaction.^27^ Due to the relatively high concentration of MAnh at the start of the reaction and the high probability of cross-propagation, the copolymer consists of long alternating Sty:MAnh sequences during the initial copolymerization period. However, as the reaction proceeds and MAnh is depleted, the probability of Sty addition increases, leading to copolymers with increased Sty fractions or even polystyrene.

In contrast, the conventional radical copolymerization of SMAnh in a CSTR, commonly employed for its commercial production, strongly reduces heterogeneity in the CCD. A CSTR operates by continuously feeding comonomers, solvent, and initiator into the reactor while simultaneously collecting the reaction mixture containing the polymer product, all under steady-state conditions.^25^ To achieve the desired statistical 2:1 Sty to MAnh copolymer, an approximated 20-fold stoichiometric excess of Sty is required throughout the reaction. Although this process allows for the production of statistical copolymers with increased homogeneity in terms of CCD, it does not eliminate all sources of chemical heterogeneity. Some variability arises from the inherent probability-based chain growth associated with conventional radical copolymerization. It is important to note that the SMA copolymers commonly used in SMALPs studies are derived from commercial SMAnh produced via the CSTR process and are typically characterized by broad MWDs and *Đ* values greater than 2.^12^

Despite extensive structural investigations supporting the current model of polymer nanodisc morphology, uncertainty remains about the exact mechanism of membrane solubilization and stabilization.^28^ The polydisperse nature of commercial SMA, in both composition and chain length, complicates the systematic evaluation of polymer-mediated membrane solubilization and hampers data interpretation. To address the limitations of commercially synthesized SMAnh, controlled radical polymerization techniques, collectively referred to as reversible deactivation radical polymerization (RDRP), have been explored.^29^ RDRP methods, such as reversible addition–fragmentation chain-transfer (RAFT)-mediated polymerization^30^ and nitroxide-mediated polymerization (NMP),^23^ enable the rational design of polymers with well-defined architectures, predetermined molecular weights, and narrow MWDs (Figure 1(C)), improving copolymer synthesis for membrane solubilization applications. RAFT-mediated polymerization (Figure 1(B)) has gained significant attention due to its effectiveness in providing living characteristics to radical polymerization. In this process, the chain transfer agent (CTA), also known as RAFT agent, regulates polymerization while offering the potential for chain-end functionality.^31^ The RAFT agent, containing a thiocarbonyl thio moiety, controls chain growth through a chain-transfer-based reversible activation/deactivation mechanism, enabling precise control over polymer chain length and therefore molecular weight. The nature of the RAFT agent is essential, as it directly affects the polymerization kinetics, chain-end functionality, and compatibility with different monomer systems.^32^ Refer to existing review articles for a deeper understanding of RDRP techniques, specifically RAFT-mediated polymerization.^19,29,33^

When MAnh is copolymerized with an excess of Sty via RAFT-mediated polymerization, an *in situ* diblock copolymer usually forms. After hydrolysis, this copolymer consists of an alternating Sty/MA block with a tail of a styrene homopolymer, known as poly­((styrene-*alt*-maleic acid)-*block*-styrene). This formation differs from the more random arrangement found in commercial SMA variants (Figure 1(D)). Smith and colleagues investigated how variations in the composition of RAFT-mediated SMA copolymers, particularly the differing amounts of alternating SMA and polystyrene blocks, impact membrane solubilization efficiency.^30^ Their research revealed that these compositional gradients significantly affect the overall solubilization efficiency, where the inclusion of polystyrene blocks was detrimental in many cases. Furthermore, polymers with low dispersities and steep gradients yielded nanodiscs of lower dispersity. This study evaluated the synthetic lipid solubilization capabilities of the RAFT-synthesized and commercial SMA variants. Additionally, they looked at their effectiveness in extracting GFP-tagged MPs from mammalian membranes.

Conversely, Hall et al. found that RAFT-synthesized SMA diblock copolymers effectively isolate MPs from *Escherichia coli (E. coli)* membranes, though to a lesser extent than commercial SMA at a 2:1 ratio, as determined through densitometric analysis.^34^ These findings support the notion that polymer efficacy in MP isolation is highly system-dependent. A deeper understanding of the interactions between the lipid composition and polymer characteristics may enhance the design of polymers tailored for specific applications.

While block and gradient SMAnh copolymers synthesized via RAFT-mediated polymerization can solubilize lipid membranes, they lack chemical homogeneity from the α- to ω-chain end due to the CCD. To remedy this, an iterative RAFT approach was investigated, yielding SMAnh with narrow MWDs and a periodic arrangement of comonomers from the α- to ω-chain end.^12^ This technique entails the continuous addition of comonomers during the reaction, combining the benefits of RDRP and CSTR. The resulting variable-length SMA (2:1) demonstrated solubilization efficiencies similar to those of commercially produced SMA (2:1). Beyond enabling diblock architectures, the RAFT method can yield copolymers featuring a hydrophobic *S*-alkyl trithiocarbonate end group. Research by Neville et al. examined how a large hydrophobic dodecylthio end group (-SC_12_) affects the behavior of SMA diblock copolymers during the formation of synthetic nanodiscs (using DMPC and DPPC) and the encapsulation of gramicidin, a model MP.^35^ They compared the behavior of SMA-SC_12_ to that of the same SMA copolymer after converting the end group to a cyanoisopropyl moiety (SMA-CN). Their results showed that SMA-SC_12_ has a tendency to self-assemble into higher-order aggregates. In contrast, SMA-CN and commercial SMA demonstrated different aggregation behaviors. Interestingly, SMA-SC_12_ showed limited solubilization of DPPC lipids, while SMA-CN and the commercial variants performed better. This research emphasizes the impact of end group variations and homoblock length on the amphiphilic balance of the polymer and the subsequent nanodisc formation.

In addition to the effects of CCD, the overall chemical composition of SMA influences its amphiphilicity. Scheidelaar et al.^11^ compared the solubilization efficiencies of various commercial SMA variants, with Sty/MA ratios ranging from 1.4:1 to 4:1. Among these, SMA (2:1) (Xiran 30010) was found to be the most effective membrane solubilizer. The collapsed coil conformation of the polymer allowed for its efficient insertion into the lipid bilayer, aided by a more uniform distribution of MA units along the polymer chain. This even distribution of MA is hypothesized to lower the charge density throughout the polymer chain, which minimizes polymer–polymer repulsion during the solubilization process. As noted, the CCD, MWD, and amphiphilicity are critical parameters influencing the solubilization efficiency of SMA variants. When investigating the impact of one SMA parameter on the lipid solubilization efficiency, other parameters must remain constant. Therefore, a variable-length SMAnh library with uniform CCDs was employed to study the effect of SMA chain length on monolayer lipid solubilization.^12,16^ Membrane disruption is initiated by the binding and insertion of SMA into the lipid bilayer.^36^ The efficiency of this initial step was independent of the SMA chain length when using an SMA library with narrow CCDs. While low molecular weight polymers showed faster solubilization kinetics than their high molecular weight counterparts, the overall extent of membrane solubilization and nanodisc formation remained unchanged. Interestingly, shorter chains from fractionated commercial SMA (Xiran 30010, 2:1 Sty-to-MAnh) were reported to enhance membrane insertion and solubilization efficiency. However, fractionation relies on solubility and does not guarantee consistent CCDs.^16^

The polymer architecture, chemical composition, MWD, and chain length of SMA can be tailored through the careful selection of the polymerization method (i.e., CSTR vs batch vs iterative), monomer feed composition (Sty-to-MAnh ratio), and polymerization mechanism (conventional vs controlled). Understanding the relationship between SMA characteristics and lipid solubilization is essential for elucidating the mechanism of nanodisc formation and its resultant properties. This knowledge not only enhances our comprehension of the behavior of the polymer but also lays the groundwork for the development of new and more effective polymers.

**Limitations of SMA**. Despite its potential, SMA exhibits inherent limitations. Under physiological conditions (pH 7.5–8.0), deprotonation of one of the carboxylic acid groups on the maleic acid (MA) repeat unit imparts hydrophilic character to the polymer.^11^ However, at low pH, protonation neutralizes the anionic charge, leading to aggregation and eventual precipitation. Additionally, in the presence of divalent salts such as Ca^2+^ and Mg^2+^, diacid repeat units of SMA can chelate with the metal ions, resulting in polymer precipitation. Furthermore, the charged polymer belt in the nanodiscs can interact electrostatically with the charges of the solubilized protein, potentially affecting the functional reconstitution of the protein.^37^

**A general approach to SMA modification**. The precursor to water-soluble SMA is SMAnh. The reactive MAnh repeat units of SMAnh can be exploited to generate various SMA copolymer derivatives. These cyclic MAnh repeat units undergo ring-opening upon treatment with nucleophiles, offering an opportunity to create new SMA derivatives with diverse properties and architectures. This modification has expanded SMA applications, making it a valuable toolkit in MP research. Over time, researchers have developed strategies to address the limitations of SMA. One approach to mitigate metal chelation by diacids in SMA has been the conversion of one or both carboxylic acid groups from MA. This is typically achieved by reacting MAnh repeat units with nucleophiles such as primary amines or alcohols. The result is a monofunctionalized MA derivative containing a carboxylic acid and either an ester linkage (for alcohols)^38,39^ or an amide linkage (for amines),^40,41^ as illustrated in Scheme 1. Subsequently, the amide functionality can be converted into an imide through thermally induced intramolecular condensation, forming a maleimide residue with enhanced thermal and hydrolytic stability.^42^

**Scheme 1:**
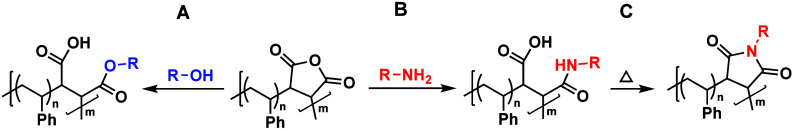
Modification of SMAnh with [A] Primary Alcohols or [B] Primary Amines and [C] Thermally-Induced Ring Closure to Form the Maleimide Residue

Furthermore, imidized copolymers can be modified with substituents bearing either a permanent charge or a pH-dependent ionizable group that undergoes deprotonation/protonation, thereby introducing the hydrophilicity required to achieve water solubility. SMAnh often serves as a starting material for derivatization. Therefore, either commercially available SMAnh variants or those synthesized through polymerization techniques such as RAFT can be used as precursor polymers. Figure 2 and Table 1 provide an overview of the next-generation SMA derivatives developed to date (Figure 2 and Table 1).

**Figure 2:**
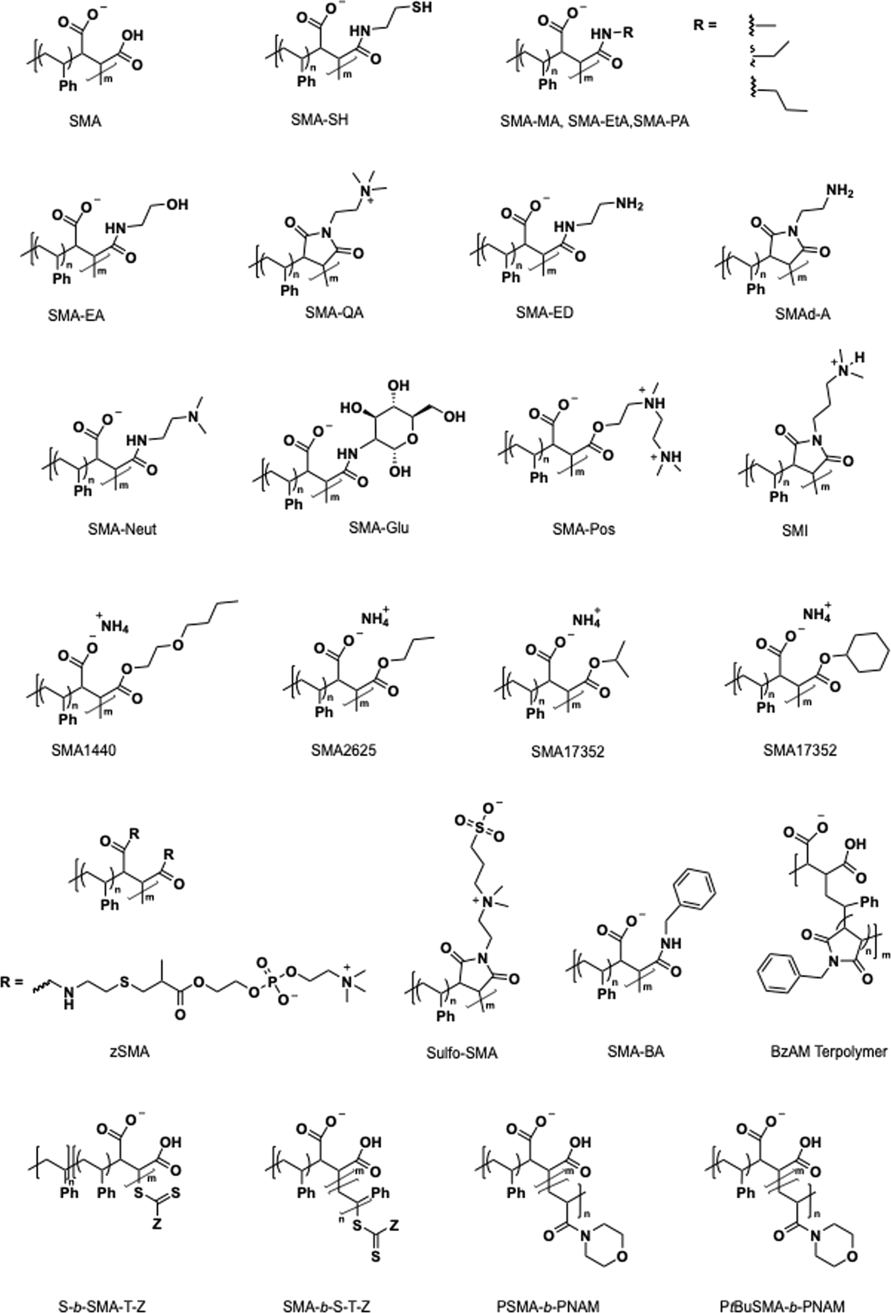
A library of available SMA derivatives.

**Table 1:** General Properties of the SMA Derivative Library

Polymer	Modification	Sty:MA Ratio	Net Charge	*M*_n_ (kDa)	*M*_w_ (kDa)	*Đ*	ref
**SMA2000**	-	2:1	Anionic	3.0	7.5	2.5	^39^
**SMA30010**	-	2.3:1	Anionic	2.5	6.5	2.6	^39^
**SMA1440**	2-Butoxyethanol	1.5:1	Anionic	2.8	7.0	2.5	^38^,^39^
**SMA2625**	*n*-propanol	2:1	Anionic	3.6	9.0	2.5	^38^,^39^
**SMA17352**	Mixture of isopropanol and cyclohexanol	1.7:1	Anionic	2.8	7.0	2.5	^38^,^39^
**SMA-MA**	*n*-Methylamine	1:1	Anionic	–	5.5	2.5	^41^,^43^
**SMA-EtA**	*n-*Ethylamine	1:1	Anionic	–	5.5	2.5	^41^
**SMA-PA**	*n-*Propylamine	1:1	Anionic	–	5.5	2.5	^41^
**SMA-Glu**	d-Glucosamine	2:1	Anionic	42.1	–	6.9	^40^
**SMA-EA**	1,2-Ethanolamine	2:1	Anionic	18.3	–	1.7	^40^
**SMA-Pos**	2-((2-(Dimethylamino)ethyl)- methylamino)ethanol	2:1	Cationic	11.1	–	1.4	^40^
**SMA-Pos**	2-((2-(Dimethylamino)ethyl)- methylamino)ethanol	3:1	Cationic	21.9	–	1.3	^40^
**SMA-Neut**	*N,N*-Dimethylethylenediamine	2:1	Zwitterionic	6.9	–	1.5	^40^
**SMA-QA**	Aminoethyl trimethylammonium chloride hydrochloride	1.3:1	Cationic	1.6	–	–	^37^,^44^
**SMAd-A**	Ethylenediamine	1.3:1	Cationic	1.6	–	–	^45^
**SMA-ED**	Ethylenediamine	1.3:1	Zwitterionic	1.6	–	–	^45^
**SMI**	*N,N*-Dimethylaminopropylamine	2:1	Cationic	2.7	7.5	2.8	^42^
**SMA-SH**	Cysteamine	2:1	Anionic	–	7.5	–	^46^
**zSMA**	Cysteamine-phosphatidylcholine	1:1	Zwitterionic	12.7–43.8	-	1.1–1.2	^47^
**Sulfo-SMA**	*N,N*-Dimethylethylenediamine +1,3-propane sultone	2:1	Zwitterionic	–	12.6	–	^48^
**SMA-BA**	*N*-Benzylamine	1:1	Anionic	2.1–7.0	2.7–9.1	1.3–1.4	^15^
**BzAM**	*N*-Benzylamine	1:1	Anionic	5.5	–	1.3	^14^
**S-** *b* **-SMA-T-Z**	Block copolymer of polystyrene and SMA	1.5:1–2.2:1	Anionic	5.3–13.2	–	1.1–1.3	^49^
**SMA-** *b* **-S-T-Z**	Block copolymer of SMA and polystyrene	1.7:1	Anionic	9.8	–	1.2	^49^
**PSMA-** *b* **-PNAM**	Block copolymer of SMA and poly(acryloyl morpholine)	1.3:1:2.4^a^	Anionic	8.3–9.4	–	1.2	^50^
**P** *t* **BuSMA-** *b* **-PNAM**	Block copolymer of *t*BuSMA and poly(acryloyl morpholine)	1.5:1:3^a^	Anionic	11.1–18.4	–	1.2–1.6	^50^

a Monomer ratios consider a 3rd monomer in the final polymer i.e. Sty:MA:3rd monomer

**Modifying SMAnh with alcohols.** Korotych et al.^39^ systematically investigated the extraction efficiency of several commercial SMA copolymers on plant thylakoid MPs. These polymers with different molecular weights and Sty-to-MA ratios included monoesterified SMA derivatives using alkoxyalcohols and aliphatic alcohols. It was shown that the 2-butoxyethanol derivative (SMA1440) had superior MP solubilization efficacy compared with its aliphatic alcohol counterparts (SMA2625 and SMA17352, Table 1). Additionally, prewashing the thylakoid membrane with EDTA reduced the effect of divalent metal ions. Although the reasons for the different solubilization behaviors of the monoesterified SMA polymers are not fully understood, the study highlights the essential role of polymer design and consequential properties in solubilization efficacy. The arrangement of monomer units and pendant groups affects the interaction of the polymer with lipid bilayers, influencing solubilization.

In a follow-up study, Hawkins et al. reported that the same monoesterified SMA derivatives exhibited no significant improvement compared to the conventional SMA (2:1) in solubilizing MPs, i.e., membrane tether protein ZipA, and the ABC transporter, BmrA, from *E. coli* membranes and the ABC transporter, MRP4/ABCC4 from Sf9 insect cells.^38^ The polymers exhibited poor stability toward Mg^2+^ at >5 mM concentration. Following Ni-NTA affinity purification, the protein yield for SMA1440 and the degree of protein purity for SMA 17352 were lower than for SMA (2:1). These findings were postulated to result from the target protein potentially binding to the resin or due to nanodisc instability caused by divalent cation sensitivity. Furthermore, purification tags (*e.g.,* His-tags) or resin binding sites may potentially interact with the charged moieties of the copolymer, thus sterically hindering or occluding the tag’s ability to bind to the affinity column.^7^ The extent of these interactions may vary in modified SMA derivatives with altered polarity and charge density.

Although the two studies yielded contrasting findings, they were conducted on distinct membrane systems. Korotych et al. examined thylakoid MP extraction using polymers with varying molecular weights and Sty-to-MA ratios, while the study by Hawkins et al. assessed the efficacy of partially esterified SMA polymers for isolating MPs from diverse membrane types compared to SMA (2:1) and SMA30010. Both studies underscore that solubilization efficacy depends on an interplay of polymer properties, membrane characteristics, and experimental conditions.

**Modifying SMAnh with amines** - *Anionic and cationic copolymers*. SMAnh copolymers functionalized with primary alkylamines revealed that the hydrophobic alkyl chain length affects solubilization efficiency and resultant nanodisc size.^41^ Using unilamellar vesicles of 1,2-dimyristoyl-*sn*-glycero-3-phosphocholine (DMPC) and the β-barrel protein PagP from *E. coli*, it was demonstrated that SMA-MA produced the smallest nanodiscs (14 nm), followed by SMA-EtA (25 nm) and SMA-PA (32 nm) at 1% (w/v) polymer concentration. An increase in the alkyl pendant length corresponded with decreased DMPC solubilization efficiency and larger nanodisc sizes, indicating an inverse relationship between the hydrophobic group length and solubilization efficacy. Despite this, all three polymer derivatives purified the β-barrel protein PagP from *E. coli* outer membrane at 2% (w/v) polymer concentration. Size-exclusion chromatography showed distinct chromatograms for each polymer, aligned with dynamic light scattering (DLS) data on nanodisc sizes. The longer-chain polymers produced two peaks, initially suggesting PagP oligomer formation; however, Western blot analysis of native and denatured fractions confirmed that the fractions contained only PagP monomers. The authors concluded that larger nanodiscs result from increased phospholipid encapsulation rather than MP aggregation.

In a separate study, Beriashvili et al. investigated SMA-MA copolymer to study calcium-dependent oligomerization of daptomycin (dap) from *Streptomyces roseosporus*, exploiting the intrinsic kynurenine fluorescence of dap for binding assays and FRET to confirm oligomer formation on nanodiscs.^43^ Dap oligomer size correlated with SMA-MA concentration, with a 9-fold SMA-MA excess producing smaller oligomers (4 subunits) than a 3-fold excess (7 subunits). Although SMA-MA nanodiscs tolerated calcium cations, they became unstable above 3 mM Ca^2+^, which is attributed to SMA-MA competing with dap for calcium chelation. This study highlights the utility of SMA-MA in the structural analysis of dap oligomers, unlike conventional SMA (2:1), due to reduced metal chelation interference.

Rothnie and co-workers^15^ evaluated SMA-benzylamine (SMA-BA) polymers produced via RAFT-mediated polymerization to achieve defined polymer lengths and a uniform distribution of hydrophobic *N*-benzylamine moieties, addressing the heterogeneity inherent to conventionally produced SMA (2:1). SMA-BA variants of three target molecular weights (i.e., 2 kDa, 4 kDa, and 7 kDa) were assessed for their membrane solubilization performance. The SMA-BA polymers effectively solubilized a range of membrane proteins of varying topologies from *E. coli, P. pastoris,* and *Sf*9 insect cells, with SMA-BA 4 kDa showing comparable purity to SMA (2:1), despite increased Mg^2+^ sensitivity.

The positively charged poly­(styrene-*co*-*N*-(3-(*N*,*N*-dimethylaminopropyl))­maleimide) (SMI) was among the first SMA derivatives to effectively solubilize human GPCRs and exhibited stability at acidic pH.^42^ Burridge et al.^40^ developed a library of SMA derivatives (SMADs) stemming from RAFT-synthesized poly­((styrene-*alt*-maleic acid)-*block*-styrene) (Figure 2) to assess the impact of polymer charge density on the reconstitution and dynamics of the human transmembrane protein KCNE1, overexpressed in *E. coli*. SMADs with negative or positive charges (SMA-Glu, SMA-EA, and SMA-Pos (2:1)) formed smaller lipid-only polymer nanodiscs, while zwitterionic and SMA-Pos (3:1) with a longer styrene tail yielded larger nanodiscs, attributed to differing surfactant-like behaviors. All SMAD-stabilized nanodiscs displayed stability across pH 3–9 and high divalent cation concentrations (<100 mM Mg^2+^), except SMA-Pos, which was insoluble around pH 5.

For spin-labeled KCNE1 reconstitution, negatively charged SMADLPs (SMA-Glu and SMA-EA) were used, and the polymer–protein interactions were evaluated using continuous wave electron paramagnetic resonance (CW-EPR) line shape analysis. SMA-Glu minimally perturbed KCNE1 dynamics, suggesting compatibility due to its lower charge density, whereas SMA-EA showed effects similar to those of conventional SMA (2:1) attributed to higher charge densities. Overall, this study underscores the influence of polymer charge density on lipid solubilization and protein stability in SMADLPs.

Ravula et al.^37^ further demonstrated this by derivatizing low molecular weight SMAnh (1.6 kDa) into positively charged SMA-QA^44^ and negatively charged SMA-EA,^51^ compared to conventional SMA (2:1). Using cytochrome P450 (positively charged) and cytochrome-*b*_5_ (negatively charged), they found that functional reconstitution of these charged MPs was enhanced when the polymer and protein charges matched, while opposing charges led to protein inactivation. These results emphasize charge consideration during polymer selection, suggesting that electroneutral polymers may allow nanodisc formation without compromising the activity of charged proteins.

In a separate study, Ravula et al. modified SMAnh (1.6 kDa) with ethylenediamine to form the zwitterionic SMA-ED and positively charged SMAd-A derivatives (Figure 1).^45^ Both SMA-ED and SMAd-A demonstrated the ability to solubilize DMPC lipids into nanodiscs that exhibited enhanced tolerance toward CaCl_2_ and MgCl_2_ (up to 200 mM). The nanodiscs of each derivative were also evaluated for their stability at pH 3–10. Nanodiscs generated by SMA-ED were stable across pH ranges except for 5–7. The authors postulated that at this pH range, the zwitterionic nature of SMA-ED induces intramolecular charge–charge interactions leading to the formation of hyper-coiling and ultimately polymer aggregation. On the other hand, nanodiscs from SMAd-A were only stable in acidic conditions (pH < 6) and precipitated above pH 6. This was attributed to the deprotonation of the ammonium cation at higher pH causing an increase in hydrophobicity. Additionally, the study demonstrated that both SMA-ED and SMAd-A could encapsulate curcumin, a hydrophobic drug, into their nanodiscs to improve its water solubility and stability. This study underscores how even a minute structural change to the polymer system can have a significant effect on its solubilization efficacy and behavior in various environmental conditions.

**Zwitterionic copolymers**. A zwitterionic SMA derivative (zSMA) was synthesized via RAFT-mediated polymerization with varying molecular weights and a Sty-to-MAnh ratio of 1:1 (Figure 2).^47^ zSMA copolymers were obtained by initially reacting cysteamine with a zwitterionic phosphatidylcholine (PC) substituent via thiol–ene chemistry. Complete functionalization of both carboxylic acid groups was achieved by coupling the cysteamine-PC intermediate to the MAnh moieties with the aid of *N,N’*-dicyclohexylcarbodiimide (DCC). The zwitterionic character was achieved above pH 5 upon deprotonation of the phosphate moiety. These zSMA-stabilized nanodiscs were stable at pH 5–10 and resistant to up to 20 mM CaCl_2_. Although zSMA shows potential, its laborious synthesis severely limits its accessibility to the biochemical community. Poly­(styrene-*co*-*N′,N′*-dimethyl­(maleimidoethyl)­ammonium propanesulfonate) (sulfo-SMA, Figure 2) is another example of an overall electroneutral polymer.^48^ Sulfo-SMA was synthetically obtained by the derivatization of SMA (2:1) with *N,N*-dimethylaminoethylamine and then reacting the resultant maleimide intermediate with 1,3-propane sultone. The zwitterionic character of sulfo-SMA emanates from the permanent positive charge of the quaternary ammonium (p*K*_a_ ∼ 10) and the negative charge of the deprotonated sulfonic acid (p*K*_a_ < 0). Similarly, zwitterionic moieties of sulfo-SMA afford solubility in aqueous media. This electroneutral polymer provides a solution to limitations associated with the high charge density of SMA, which may cause electrostatic interference in the functional reconstitution of MPs.^37,48^ Sulfo-SMA extends the utility of SMALP technology by using modern bioanalytical techniques, such as microfluidic diffusional sizing, which measures the binding of proteins to the lipid bilayer. Additionally, sulfo-SMA is amenable to new applications, such as cell-free protein synthesis allowing for the precise control of Mg^2+^ concentrations during cell-free protein translation.^48^

**Design of a SMA (2:1) mimic**. A key factor influencing the solubilization efficiency of SMA copolymers is the Sty-to-MA ratio. The SMA (2:1) variant is considered the gold standard, as it maintains an optimal balance between its hydrophobic and hydrophilic components.^34^ Consequently, SMA (2:1) is often used as the starting point for further modifications and as a control in comparative studies. However, the RAFT-mediated polymerization of Sty and MAnh is most effective for the synthesis of an alternating SMAnh (1:1) copolymer with a defined molecular weight and narrow MWD. In its water-soluble form, SMA (1:1) is considered too hydrophilic for efficient membrane insertion and solubilization.^18^ To address this issue, the overall hydrophobicity of the copolymer can be increased by functionalizing the MAnh units with aliphatic or aromatic substituents.

Kuyler et al.^14^ recently introduced a systematically designed series of poly­(styrene-*co*-maleic acid-*co*-(*N*-benzyl)­maleimide) (BzAM) terpolymers exhibiting tunable amphiphilicity through the incremental modification of SMAnh (1:1) with the styrene analogue, *N*-benzylamine. This approach enabled the precise variation of a single parameter to investigate structure–activity relationships. The terpolymer system was designed to mimic the solution properties and solubilization performances of the conventional SMA (2:1) copolymer while offering enhanced control over the molecular weight and distribution. By modulating the hydrophobicity across the BzAM terpolymer series, the study established a versatile platform for systematic investigations, demonstrating that increased hydrophobicity correlated with improved solubilization yields of Sav1866, a 12-transmembrane ABC transporter isolated and purified from *E. coli* membranes.

**Conjugating biomolecules to SMA**. The SMALP technology significantly simplified the extraction of MPs from the lipid bilayer. However, subsequent protein purification steps often require genetic engineering that could disrupt the structural integrity of the protein. This is often accompanied by the incorporation of functional compounds (tags) to the protein of interest to enhance its detection.^46^ To expand the scope of functionalized polymers for MP research, polymers can be strategically designed to contain specific functionalities (*e.g*., thiol, azide, and alkyne) that will facilitate the attachment of fluorescent or affinity tags with utility in protein purification and downstream applications.

For example, Lindhoud et al.^46^ modified commercial SMAnh (2:1) with cysteamine at different SMAnh to cysteamine molar ratios (*i.e*., 1:1, 1:2 and 1:3) to produce a sulfhydryl-containing SMA (SMA-SH). These SMA-SH polymers consisted of various amounts of free thiols randomly grafted along the polymer backbone, which facilitated the bioconjugation of thiol-reactive probes such as green-fluorescent dye (Alexa Fluor 488) and maleimide-biotin using thiol–ene “click” chemistry. The inclusion of a fluorophore allows for the application of the FRET technique to demonstrate the successful functionalization of SMA-SH. The FRET phenomenon is based on the transfer of excitation energy from a donor fluorophore to an acceptor chromophore when they are in close proximity. Biotin is a small biomolecule with a strong binding affinity for avidin protein. This biotin–avidin interaction has been vastly used in biological applications, including the immobilization of biomolecules on surfaces, enabling the study of protein dynamics. One of the advantages of SMA-SH is that its synthesis is facile and cost-effective, making it universally accessible to bio/chemical laboratories.

Additionally, due to its thiol groups, SMA-SH is a versatile tool compatible with various thiol-reactive probes, allowing researchers to fine-tune it for specific applications. Covalently attached biotin or a fluorophore opens new doors for biophysical and biochemical analytical tools to elucidate protein structures. One potential drawback of the SMA-SH approach is the random distribution of thiol functionalities along the backbone. With a typical molar mass of 5 kDa, SMAnh (1:1) contains on average 25 MAnh repeat units. If 1% of those are modified with cysteamine, on average, one in four chains will have a thiol functionality. However, due to the statistical nature of the modification, there may be chains that contain more than one and certainly a large fraction of chains that contain none. An alternative approach to explore is the introduction of a thiol at one of the polymer chain ends, which can be achieved through living polymerization techniques, *e.g.,* RAFT-mediated polymerization. This will provide a better defined system with one thiol functionality per chain and always placed at the chain end.

**Block copolymers (BCPs)**. One of the properties of RDRP techniques is the generation of macromolecular architectures with increased complexity, such as block copolymers (BCPs). BCPs are polymer chains composed of two or more distinct sequences or “blocks” containing either different monomers or monomer sequence distributions (See Figure 1(D)). In contrast to RDRP, conventional radical polymerization is incapable of generating such structures.

The recent implementation of BCPs within the polymer nanodisc field has demonstrated promising potential for applications involving the immobilization of lipid nanodiscs. Research by Farrelly et al. explored the use of BCPs with distinct architectures, i.e., ((Sty)-*b*-(SMA)-T-Z (hydrophobic–hydrophilic) and (SMA)-*b*-(Sty)-T-Z (hydrophilic–hydrophobic)) to tether lipid nanodiscs onto gold surfaces.^49^ Enhanced DMPC solubilization was observed with BCPs containing a more hydrophilic Z-group, shorter polystyrene block, and a lower overall molecular weight.^49^ Furthermore, the BCPs were found to bind to the gold surface as a viscoelastic film irrespective of the presence or absence of the trithiocarbonate end group. Nevertheless, BCPs with trithiocarbonate end groups exhibited increased gold affinity, especially when the trithiocarbonate end group carried a hydrophilic substituent.^49^ The authors noted that the hydrophobic polystyrene block induced an increase in the adsorbed mass onto the gold surface. This indicates that binding of lipid nanodiscs to the gold surface proceeds via a variety of interactions besides those of the trithiocarbonate moiety.^49^

A recent study by Ball et al. replaces the hydrophobic spacer with a more hydrophilic block, utilizing double hydrophilic block copolymers (DHBCs) composed of SMA/*t*BuSMA and 4-acryloylmorpholine (NAM).^50^ The incorporation of NAM as a second block to SMA/*t*BuSMA afforded polymers capable of DMPC solubilization at Mg^2+^ concentrations of 0–100 mM (P*t*BuSMA-*b*-PNAM), and 4–100 mM (PSMA-*b*-PNAM); and Ca^2+^ concentrations of 0–8 mM (P*t*BuSMA-*b*-PNAM), and 4–8 mM (PSMA-*b*-PNAM).^50^ The lipid nanodiscs formed with DHBCs are stable at higher cation concentrations with only slight hydrodynamic diameter increases for P*t*BuSMA-*b*-PNAM (9–21 nm, 0–10 mM Mg^2+^ and 21–26 nm, 10–400 mM Mg^2+^) and PSMA-*b*-PNAM (26–42 nm, 0–40 mM Ca^2+^ and 42–67 nm, 40–400 mM Ca^2+^), where *t*BuSMA (non-BCP) aggregated at low Mg^2+^ and Ca^2+^ concentrations (∼2 mM). The increased stability at higher cation concentrations is most likely due to steric stabilization of the nanodiscs, as opposed to electrostatic stabilization.

These cation-tolerant DHBCs could afford surface tethering of lipid nanodiscs with limited nonspecific surface deposition, therefore encouraging the tethering of lipid nanodiscs to gold surfaces via the trithiocarbonate moiety. This approach may enable future applications in surface-based techniques such as surface plasmon resonance (SPR) to study MPs in a more native-like lipid environment.

**Alternative polymer systems**. In recent years, alongside efforts to modify the architecture of conventional SMA and develop various derivatives, there has been an increasing interest in alternative polymer systems for MP solubilization. These alternative approaches aim to expand the repertoire of polymers available for MP research. A defining feature common to these polymers is their amphiphilic nature, which plays a crucial role in the formation of polymer-stabilized nanodiscs. Ongoing research continues to expand the utility of amphiphilic polymers that demonstrate efficacy in MP solubilization. Each polymer system presents distinct advantages and drawbacks with selecting an appropriate polymer dependent on the characteristics of the membrane environment, the target protein, and downstream processing requirements. Herein, we review four leading SMA alternatives developed to date, as illustrated in Figure 3, and the general properties of those are summarized in Table 2.

**Figure 3:**
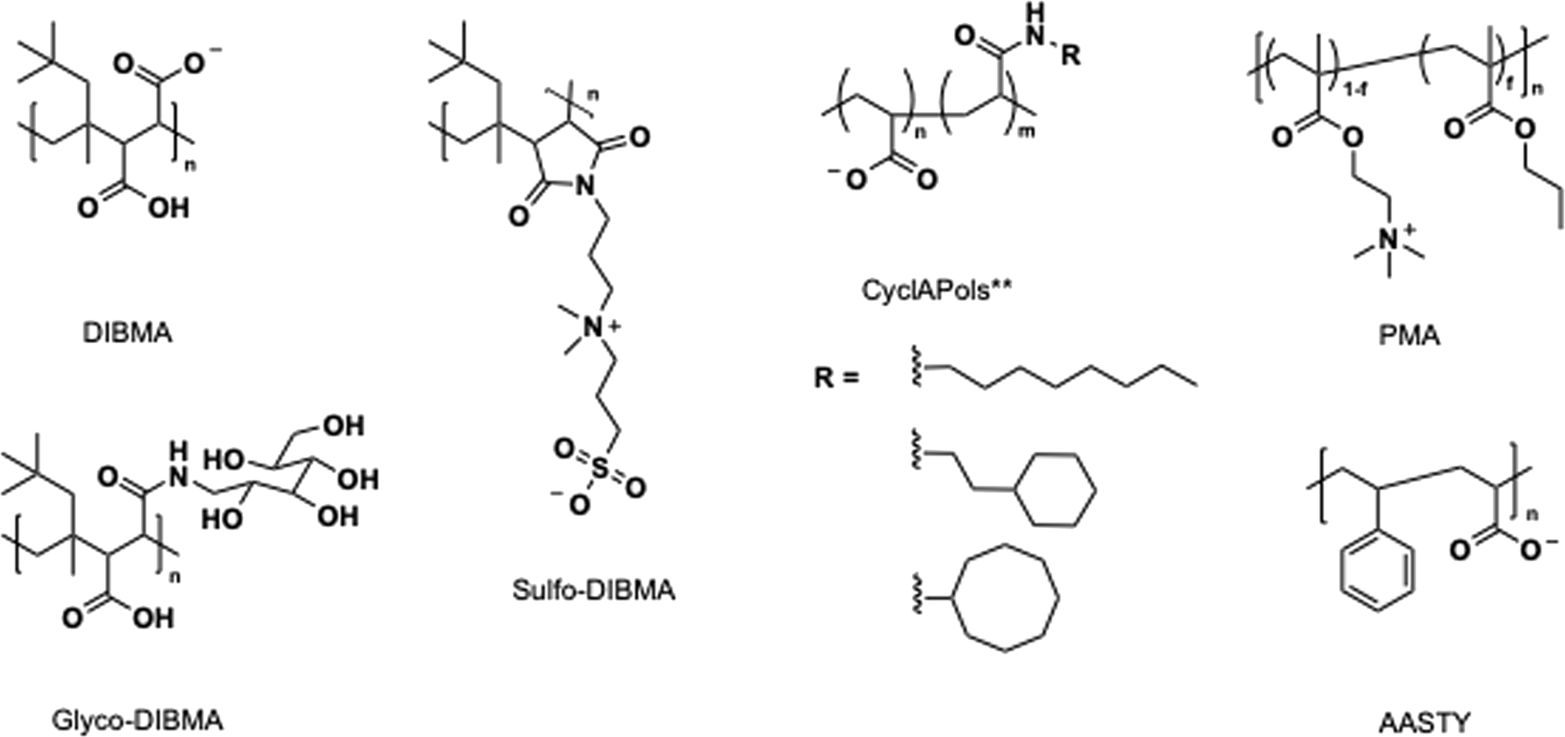
A library of SMA alternatives.

**Table 2:** General Properties of SMA Alternatives

Polymer	A:B Ratio	A (Hydrophobic)	B (Hydrophilic)	Net charge	*M*_n_^a^ (kDa)	*M*_w_^a^ (kDa)	*D̵*	ref
**DIBMA**	1:1	Diisobutylene	Maleic acid	Anionic	8.4	15.3	1.8	^52^,^53^
**Glyco-DIBMA**	1:1	Diisobutylene	Maleic acid modified with *N*-methyl-*D*-glucamine	Neutral	–	10.0^b^	–	^54^
**Sulfo- DIBMA**	1:1	Diisobutylene	Maleic acid modified with *N,N-*dimethylamino-propylamine +1,3-propanesultone	Zwitter-ionic	8.4	15.3	1.8	^48^
**CycloApols**	1:1	Cyclic substituted acrylamides	Acrylic acid	Anionic	2.8	5.5	2.0	^55^,^56^
**PMA**	1.5:1	Butyl methacrylate	Methacroylcholine chloride	Cationic	11.8^c^	–	–	^57^,^58^
**AASTY**	1.3:1	Styrene	Acrylic acid	Anionic	5.5	6.6	1.2	^59^,^60^

a Molecular weight of base polymers.

b As reported by *Dow Inc.*. for their ACUSOL 460ND.

c Calculated from the DP reported by *Avanti Research* for PMA N–C4–52–6.9.

**Poly­(diisobutylene-alt-maleic acid) and its derivatives**. One of the most extensively reported non-SMA-based amphiphilic copolymers for MP solubilization is poly­(diisobutylene-*alt*-maleic acid) (DIBMA). Unlike SMA, which incorporates aromatic styrene groups, DIBMA features short-chain branched aliphatic diisobutylene units, providing distinct physicochemical properties. Both SMA and DIBMA are typically synthesized *via* conventional radical polymerization,^52^ with the commercial DIBMA variant derived from Sokalan CP9 (BASF, Germany) exhibiting a broad MWD. However, DIBMA can also be synthesized *via* RAFT-mediated polymerization, enabling narrower MWDs and offering the possibility to introduce chain-end functionalities.^13^

DIBMA lipid particles or DIBMALPs have demonstrated potential advantages over SMA. Oluwole et al.^53,61^ were the first to use DIBMA for MP solubilization. They showed its effectiveness across phospholipids of varying chain lengths, including 1,2-dimyristoyl-*sn*-glycero-3-phosphocholine (DMPC), 1,2-dipalmitoyl-*sn*-glycero-3-phospho-choline (DPPC), and 1,2-dilauroyl-*sn*-glycero-3-phosphocholine (DLPC).^53^ Notably, DIBMALPs showed enhanced stability in the presence of divalent cations, withstanding up to 20 mM of Ca^2+^ or Mg^2+^, compared to SMALPs, which precipitated at just 2 mM.^53^ Interestingly, DIBMALPs were larger than corresponding SMALPs, as determined by DLS analysis.^62^

Gulamhussein et al.^54^ investigated the solubilization efficiency of DIBMA for various MPs. They assessed its performance with the membrane tether protein ZipA (*E. coli*), ABC transporter BmrA (*E. coli*), and the G-protein-coupled receptors (GPCRs) adenosine A2a receptor (A_2A_R) expressed in *Pichia pastoris* and the calcitonin gene-related peptide (CGRP) receptor expressed in Cos7 cells. While SMA outperformed DIBMA for bacterial MPs such as ZipA and BmrA, DIBMA effectively solubilized comparable levels of functional GPCRs from eukaryotic cells. Yet, the SMALPs exhibited increased stabilities compared to DIBMALPs. The authors postulated that the phenyl groups of SMA, known to insert into the core of the bilayer, increase the rigidity of the lipids in the outer layer. The branched aliphatic pendant groups of DIBMA have a milder impact on lipid acyl chain order.^53,54,61^ Furthermore, the aliphatic diisobutylene units of DIBMA mitigate interference with optical spectroscopic techniques in the far-UV range, offering a distinct advantage for certain applications.^53,54,61^

Recent efforts have focused on developing DIBMA derivatives to enhance the performance further. Glyco-DIBMA, introduced by Danielczak et al.,^63^ features partial amidation with the amino sugar *N*-methyl-d-glucamine, increasing overall hydrophobicity and reducing charge density. This derivative forms smaller, more uniform nanodiscs than DIBMA. Using DMPC lipids at a polymer-to-lipid mass ratio of 1.5, the hydrodynamic diameters of DIBMALPs and Glyco-DIBMALPs were approximately 31 ± 13 and 15 ± 6 nm, respectively. Furthermore, Glyco-DIBMA demonstrated improved efficiency in solubilizing bacterial MPs, particularly at higher polymer concentrations. Glyco-DIBMALPs also support protein-conjugation techniques, making them well-suited for advanced *in vitro* applications.

Sulfo-DIBMA, a zwitterionic modification analogous to Sulfo-SMA, incorporates a quaternary ammonium and sulfobetaine group to neutralize charge density.^48^ As a result, Sulfo-DIBMALPs exhibit enhanced stability in the presence of high concentrations of divalent cations (up to 80 mM). Sulfo-DIBMA demonstrated compatibility with cell-free membrane protein translation and cryo-EM studies. Moreover, it preserved charge-sensitive interactions between proteins and lipids, allowing these interactions to be effectively detected using microfluidic diffusional sizing.

**Amphipol derivatives**. Amphipols (APols) are statistical copolymers composed of acrylic acid and *N-*substituted acrylamides, resulting in a highly hydrophilic backbone grafted with hydrophobic side chains, which gives rise to its amphiphilicity. Introduced by Tribet et al.^64^ in 1996, APols were designed as a new class of polymers to maintain the water solubility of MPs in the absence of free detergent. Conventional APols, based on poly­(acrylic acid) (PAA) derivatives, seldom facilitate the direct extraction of MPs from biological membranes since the MP must first be transferred from the detergent-solubilized extract.^55,64^ A significant drawback of this membrane-mimetic system is the lack of a native-like lipid bilayer surrounding the isolated MP.

Marconnet et al. and Higgins et al.^56,57^ synthesized PAA derivatives comprising cyclic rather than linear aliphatic side groups, referred to as cycloalkane-modified amphipols (CyclAPols). These were the first amphipol-based polymers capable of solubilizing and stabilizing MPs in a single step without detergents. Similar to the modifications seen in SMAnh copolymers, CyclAPols (and other APols) can be functionalized with different groups, expanding their potential applications. This versatility makes them useful tools for biochemists and structural biologists to extract, handle, and study MPs.

The solubilization efficiency of CyclAPols was compared to that of other polymers for extracting MPs from *E. coli* membranes and the native purple membrane of *Halobacterium salinarum*.^56^ CyclAPols were found to solubilize the bacteriorhodopsin (BR) protein from the purple membrane (HsBR), which is known for its resistance to solubilization.

Furthermore, CyclAPols have proven to be compatible with UV–visible spectroscopy and have been successfully used in structural studies of MPs, including cryo-EM single-particle analysis and electrospray ionization mass spectrometry.^57^

**Polymethacrylates**. Prior to the development of CyclAPols, conventional amphipols inspired another amphiphilic copolymer for MP solubilization. In 2017, Yasuhara et al.^58^ reported the synthesis of a library of polymethacrylate (PMA) random copolymers as alternatives to SMA. The copolymers consisted of hydrophobic butyl methacrylate and hydrophilic methacroylcholine chloride monomers. Today, a variety of methacrylate monomers can be incorporated into PMA. The composition ratio of the two monomeric components can also be varied over a wider range than that of SMA or DIBMA because the monomers do not exhibit the same tendency to produce alternating copolymers. These monomers are relatively inexpensive and allow for cost-effective, large-scale production.

The PMA series prepared by Yasuhara et al.^58^ was able to form DMPC lipid-only nanodiscs of approximately 17 nm, as determined by cryo-TEM. Additionally, PMA-stabilized lipid nanodiscs were used to reconstitute and stabilize a helical structural intermediate state of a human islet amyloid polypeptide (hIAPP) inhibiting aggregation and the formation of beta-amyloid fibrils. Their study did not explicitly measure the cation tolerance of their PMA nanodiscs; however, many of their analyses were successfully carried out in the presence of Ca^2+^ (2 mM) and Mg^2+^ (10 mM). Furthermore, Lavington et al.^65^ demonstrated the detergent-free solubilization and purification of a class A GPCR, NTSR1, from biological membranes using PMA. The compatibility of PMA with standard purification protocols and millimolar concentrations of divalent cations suggest a potentially broad scope for PMA in solubilizing MPs such as GPCRs. PMA lacks a light-absorbing aromatic group and is, therefore, compatible with circular dichroism, UV–visible, and fluorescence spectroscopy experiments.^59^

**Poly­(acrylic acid-*co*-styrene)**. Poly­(acrylic acid-*co*-styrene) (AASTY) has only recently emerged as a suitable replacement for SMA that can be employed for MP solubilization. This was first demonstrated by Smith et al.^66^ in 2020. They showed that AASTY can solubilize the human transient receptor potential melastatin type 4 (hTRPM4) from mammalian membranes and that it holds promise in single-particle cryo-EM on human integral MPs.

AASTY is a copolymer like SMA, except that the MA groups are replaced by acrylic acid (AA) groups. These structural differences translate into anionic AASTY exhibiting a slight reduction in sensitivity to divalent cations compared to diacid-containing SMA.^60^ AASTY is still susceptible to similar charge-related limitations experienced by SMA. In a recent study, Timcenko et al.^67^ evaluated the interactions between divalent cations and AASTY nanodiscs. In comparison to SMA (2:1), AASTY exhibited comparable tolerances toward Ca^2+^ (5 mM) and increased stability toward Mg^2+^ (15 mM).

Similar to alternating SMA, AASTY is often synthesized *via* RAFT-mediated polymerization for enhanced control over the molecular weight, dispersity, and monomer gradient within the copolymer. It is also thought to exhibit an alternating comonomer distribution. However, Kopf et al.^68^ hypothesized that AASTY likely has less alternating character compared to SMA. The reactivity ratios reported in the following studies were calculated using the Fineman–Ross method and can be used to explain this prediction. Baruah et al.^69^ reported the reactivity ratios for Sty and MAnh to be 0.121 and 0.009, while Chernikova et al.^70^ reported the reactivity ratios for Sty and AA to be 1.01 and 0.16. Although these values are influenced by various experimental conditions, the distinct difference is the near zero reactivity ratio for MAnh during the copolymerization of SMAnh. This implies that no two MAnh units will insert next to each other. Furthermore, the relatively small reactivity ratio of Sty leads to predominantly alternating monomer insertions for SMAnh. The reactivity ratio of AA during the copolymerization of AASTY is also small but still not close to zero. Therefore, the probability of homopropagation (of either type of monomer) is much higher during the copolymerization of Sty and AA, and AASTY will be “less alternating” than SMA. Consequently, the preparation of AASTY variants with diverse compositions is more readily achievable compared to SMA.^71^ Another similarity between AASTY and SMA is the presence of aromatic Sty groups, making it incompatible with far-UV optical spectroscopy.

**Applications of SMAnh copolymers**. SMAnh copolymers synthesized through conventional radical polymerization have been used industrially for many years and have found use in a wide range of applications, including paper-making,^24^ builder systems of carpet cleaners,^72^ thiadiazole-modified SMA for energy storage,^73^ and many more. One of the most significant features of SMA is its ability to adopt a secondary structure in which the hydrophobic and hydrophilic functionalities occupy different regions and form an amphiphilic assembly. Consequently, combining this polymer with a phospholipid bilayer leads to the spontaneous formation of polymer–lipid nanostructures in which the polymer surrounds the acyl chains of lipids like a “bracelet”.^5^ This principle was first applied by Knowles et al.^6^ to isolate MPs in a nanoparticle consisting of lipid bilayer and SMA (2:1). Since then, SMALPs have found an array of applications, particularly within biological fields, where the isolation of MPs is a vital step in many analyses. One of the main advantages of using amphiphilic polymers for MP isolation is the retention of the structure and conformation of the protein, allowing the study of conformational changes in dynamic proteins in response to stimuli. This was demonstrated by Grime et al.^74^ when the GPCR rhodopsin was isolated using SMA, SMI, and DIBMA. The change in retinal conformation, the photoreactive chromophore of rhodopsin, from the *cis*- to the *trans*-configuration, was monitored as this drives rhodopsin from the inactive dark state to the fully active Meta II state. This transition from the inactive state up to the intermediate Meta I state causes no significant structural changes in the protein, but transitioning from Meta I to Meta II is accompanied by a large conformational change through the movement of the transmembrane helix-6. It was found that SMA- and SMI-encapsulated rhodopsin allowed for only transition to the intermediate Meta I state, while DIBMA-encapsulation allowed for full transition to the Meta II state. This demonstrates the importance of the correct polymer selection for MP isolation, as variations in polymer properties may influence the conformational state of the polymer-stabilized MP.

Zhou et al.^75^ conducted a study utilizing SMALPs to investigate protein conformation, specifically examining the envelope glycoprotein trimer (Env) of the human immunodeficiency virus (HIV). Env was incorporated into SMALP nanodiscs and exposed to both poorly neutralizing antibodies (pNAbs) and broadly neutralizing antibodies (bNAbs). Since the recognition of certain bNAbs depends on the conserved pretriggered State-1 conformation of Env, preserving this structural state is crucial for binding. To assess stability, the wild-type HIV-1_AD8_ and 2–4 RM6 Env trimers were solubilized using SMA, the nonionic detergent Cymal-5, or a simple buffer solution (RIPA), and their integrity was monitored over time and across varying temperatures. The results demonstrated that SMA provided superior stabilization of Env, maintaining trimer integrity more effectively at room temperature.

Another interesting application of SMA-like polymers was demonstrated by Buachi et al.^76^ in encapsulating propolis, a natural product used in therapeutics for its many beneficial properties, including antimicrobial, anti-inflammatory, and wound-healing properties.^77^ Propolis extraction is usually performed in weakly polar organic solvents, as natural compounds possessing antioxidant activity (such as flavonoids) are not found in the aqueous extraction fraction. These solvents have undesirable effects, such as skin irritation and toxicity, necessitating alternative flavonoid extraction methods. Nanoencapsulation of propolis in SMALPs increases bioavailability and promotes cell growth when administered at low concentrations. SMA provides a potential solution for a critical challenge faced in drug delivery: incompatibility in the hydrophobic/hydrophilic balance between drugs and biological systems. One example of this issue was studied by Torgersen et al.^78^ when using SMALPs for the delivery of the anticancer drug doxorubicin (DOX), a molecule with low water solubility. DOX-containing lipid nanoparticles were taken up into cells and degraded by lysosomes, leading to the release of DOX and, consequently, a reduction in the cell proliferation of several cancer cell lines. The biodistribution of the nanoparticles indicated that the highest uptake was in the liver, with a high rate of excretion into the intestine through the bile, suggesting that this may be a useful system for drug delivery.

**Molecular dynamic simulations for predictive work**. Computational chemistry has emerged as a powerful tool for exploring polymer properties relevant to membrane solubilization, complementing experimental approaches. Advances in molecular modeling have enabled the detailed investigation of membrane systems at both molecular and atomistic levels, offering unique insights that enhance the interpretation of experimental results.^79–82^ Previous studies have demonstrated the utility of computational techniques in membrane research, reinforcing or validating hypotheses based on experimental findings.^83–85^ Schmidt et al. utilized coarse-grain molecular dynamics simulations to gain insights into the lipid–protein interactions surrounding Aquaporin Z (AqpZ).^83^ Their simulations identified a specific cardiolipin binding site at the interface of the AqpZ tetramer, which is consistent with findings from mass spectrometry. The computational analysis also revealed a distinct lipid composition in the AqpZ membrane environment, showing a notable depletion of cardiolipin compared to the inner membrane of *E. coli*. Instead, anionic lipids, including cardiolipin, preferentially localized near the protein surface, a conclusion supported by lipidomic analysis indicating phosphatidylethanolamine enrichment in the lipid annulus surrounding AqpZ. Additionally, molecular dynamics simulations shed light on the hydrophobic mismatch between AqpZ and the lipid membrane. The simulations provided a rationale for experimental observations, suggesting that the high entropic cost of lipid sorting in the fluid membrane contributed to this mismatch.

Colbasevici et al.^84^ combined computational analysis with electron paramagnetic resonance (EPR) experiments to investigate the interactions of SMA and DIBMA copolymers with nanodiscs formed from synthetic membranes of DMPC and POPC. Experimental findings revealed that lipids within DIBMA-stabilized nanodiscs displayed greater flexibility than those stabilized by SMA. Molecular dynamics simulations supported these observations, showing weaker interactions between DIBMA and the encapsulated lipids, resulting in a more fluid lipid environment. EPR spectroscopy further highlighted differences in nitroxide spin label motion, with spin labels in SMA-stabilized nanodiscs exhibiting more restricted movements than those in DIBMA-stabilized counterparts. Simulations provided quantitative data on the order parameters and rotational correlation times, corroborating the experimental results. These findings underscore the synergy between experimental and computational approaches, where simulations can offer insights into chemical processes. As computational power continues to advance, it holds the potential to drive intelligent polymer design, enabling more effective membrane solubilization strategies.

Achieving the optimal balance between hydrophobic and hydrophilic properties is crucial when designing amphiphilic polymer systems for efficient solubilization. Incorporating computational models into the prescreening process can predict and optimize the relative amphiphilicity of a given polymer system, providing valuable guidance for the targeted design and synthesis of novel systems. Establishing a standardized protocol for molecular modeling within the SMALP community would further enhance these efforts. Such a protocol, compatible with various computational software packages, would improve accessibility and enable researchers to make informed decisions regarding polymer design. This approach can potentially accelerate the development of advanced polymer systems, conserve chemical resources, and reveal trends in the polymer architecture for membrane solubilization.

Molecular dynamics simulations hold great promise for advancing our understanding of nanodisc formation in polymer-mediated membrane solubilization. Detailed insights into this process could enable the design of polymers tailored explicitly for different applications. Currently, membrane solubilization is thought to occur in three stages: polymer binding to the membrane surface, polymer insertion and membrane disruption, and nanodisc formation. Orekhov et al.^86^ comprehensively reviewed this mechanism, while Xue et al. (Figure 4)^36^ modeled the membrane solubilization process mediated by SMA. Despite being a coarse-grain model, it offered insights into the mechanism of polymer insertion, suggesting that the Sty unit of SMA initiates membrane insertion.

**Figure 4:**
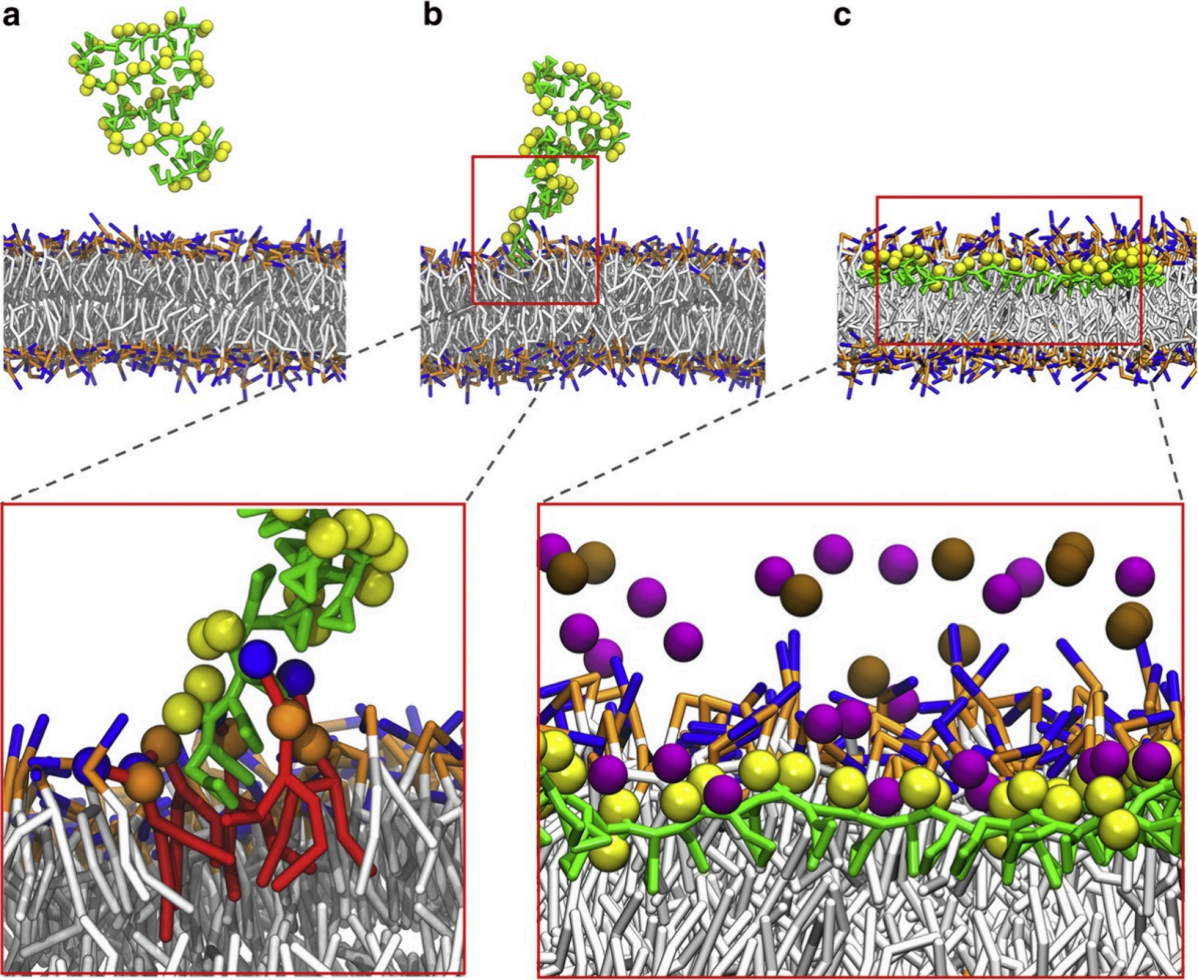
Membrane solubilization mechanism of SMA. Reproduced from Xue et al.^36^

Future studies should aim to uncover the mechanistic details of how amphiphilic polymers solubilize and stabilize MPs, potentially identifying distinct modes of polymer insertion that account for variations in solubilization kinetics. Molecular dynamics simulations could guide the design of polymers with tailored insertion rates to meet specific system requirements. This approach could also facilitate screening novel polymers, predicting their insertion behavior and rates in different membrane environments, further advancing the development of targeted polymer systems.

## Conclusions and Future Outlook

The development and application of amphiphilic polymers, particularly in the formation of polymer-stabilized nanodiscs, have significantly advanced membrane protein (MP) research. These polymers provide a robust and versatile method for the extraction, purification, and stabilization of MPs in their native lipid environments, facilitating detailed structural and functional analyses. Despite the substantial progress, challenges persist, including the susceptibility of most SMA-based polymers to divalent cations and limitations in compatibility with certain spectroscopic analyses.

Ongoing research aims to address these challenges through the design and synthesis of next-generation polymers with enhanced properties. Innovations such as controlled polymerization techniques and the development of alternative polymers like DIBMA and poly­(acrylic acid)-based materials offer promising solutions to overcome the constraints of conventional SMA.

Future research could focus on key aspects of the technology and the polymers that facilitate it. Detailed mechanistic studies should be prioritized to deepen our understanding of membrane solubilization and nanodisc formation processes. Such insights could guide the targeted design of polymers tailored to distinct applications. However, with or without input from computational studies, it is important to continue exploring and refining the design of polymers that offer improved stability and compatibility with various biophysical and biochemical techniques. To that end, it is crucial to build an increased understanding of structure–activity relationships for various polymers. Ideally, these relationships should be developed based on a carefully designed series of polymers that vary in just one parameter within the series.

Beyond their established role in MP isolation and stabilization for fundamental research and drug discovery applications, polymer-stabilized nanodiscs hold the potential for broader applications, such as drug delivery. Their nanoscale size, significantly smaller than that of conventional polymeric drug delivery systems like vesicles and nanoparticles, positions the polymer nanodisc technology as a promising candidate for innovative therapeutic strategies. As the field continues to evolve, the versatility and impact of amphiphilic polymers are expected to extend into new and transformative domains.

## References

[1] Hedin L. E., Illergard K., Elofsson A. (2011). An introduction to membrane proteins. J. Proteome Res..

[2] Santos R., Ursu O., Gaulton A., Bento A. P., Donadi R. S., Bologa C. G., Karlsson A., Al-Lazikani B., Hersey A., Oprea T. I., Overington J. P. (2017). A comprehensive
map of molecular drug targets. Nat. Rev. Drug
Discovery.

[3] Van
Meer G., Voelker D. R., Feigenson G. W. (2008). Membrane lipids: where they are and
how they behave. Nat. Rev. Mol. Cell Biol..

[4] Seddon A. M., Curnow P., Booth P. J. (2004). Membrane
proteins, lipids and detergents:
not just a soap opera. Biochim. Biophys. Acta
- Biomembr..

[5] Tonge S., Tighe B. (2001). Responsive hydrophobically associating
polymers: a review of structure
and properties. Adv. Drug Delivery Rev..

[6] Knowles T. J., Finka R., Smith C., Lin Y.-P., Dafforn T., Overduin M. (2009). Membrane proteins solubilized intact in lipid containing
nanoparticles bounded by styrene maleic acid copolymer. J. Am. Chem. Soc..

[7] Lee S. C, Knowles T. J, Postis V. L G, Jamshad M., Parslow R. A, Lin Y.-p., Goldman A., Sridhar P., Overduin M., Muench S. P, Dafforn T. R (2016). A method
for detergent-free isolation
of membrane proteins in their local lipid environment. Nat. Protoc..

[8] Pollock N. L., Lee S. C., Patel J. H., Gulamhussein A. A., Rothnie A. J. (2018). Structure and function of membrane
proteins encapsulated
in a polymer-bound lipid bilayer. Biochim. Biophys.
Acta - Biomembr..

[9] Sun C., Gennis R. B. (2019). Single-particle cryo-EM studies of transmembrane proteins
in SMA copolymer nanodiscs. Chem. Phys. Lipids.

[10] Ayub H., Murray R. J., Kuyler G. C., Napier-Khwaja F., Gunner J., Dafforn T. R., Klumperman B., Poyner D. R., Wheatley M. (2024). GPCRs in the round: SMA-like copolymers
and SMALPs as a platform for investigating GPCRs. Arch. Biochem. Biophys..

[11] Scheidelaar S., Koorengevel M. C., van Walree C. A., Dominguez J. J., Dörr J. M., Killian J. A. (2016). Effect of polymer composition and
pH on membrane solubilization by styrene-maleic acid copolymers. Biophys. J..

[12] Cunningham R. D., Kopf A. H., Elenbaas B. O., Staal B. B., Pfukwa R., Killian J. A., Klumperman B. (2020). Iterative
RAFT-mediated copolymerization
of styrene and maleic anhydride toward sequence-and length-controlled
copolymers and their applications for solubilizing lipid membranes. Biomacromolecules.

[13] Ball L. E., Riley L. J., Hadasha W., Pfukwa R., Smith C. J., Dafforn T. R., Klumperman B. (2021). Influence
of DIBMA polymer length
on lipid nanodisc formation and membrane protein extraction. Biomacromolecules.

[14] Kuyler G. C., Barnard E., Sridhar P., Murray R. J., Pollock N. L., Wheatley M., Dafforn T. R., Klumperman B. (2025). Tunable Terpolymer
Series for the Systematic Investigation of Membrane Proteins. Biomacromolecules.

[15] Akram A., Hadasha W., Kuyler G. C., Smith M.-P., Bailey-Dallaway S., Preedy A., Browne C., Broadbent L., Hill A., Javaid T. (2025). Solubilisation &
purification
of membrane proteins using benzylamine-modified SMA polymers. Biophys. Chem..

[16] Dominguez
Pardo J. J., Koorengevel M. C., Uwugiaren N., Weijers J., Kopf A. H., Jahn H., van Walree C. A., van Steenbergen M. J., Killian J. A. (2018). Membrane solubilization by styrene-maleic
acid copolymers: delineating the role of polymer length. Biophys. J..

[17] Morrison K. A., Akram A., Mathews A., Khan Z. A., Patel J. H., Zhou C., Hardy D. J., Moore-Kelly C., Patel R., Odiba V. (2016). Membrane protein extraction
and purification using styrene–maleic acid (SMA) copolymer:
effect of variations in polymer structure. Biochem.
J..

[18] Swainsbury D. J., Scheidelaar S., Foster N., Van Grondelle R., Killian J. A., Jones M. R. (2017). The effectiveness of styrene-maleic
acid (SMA) copolymers for solubilisation of integral membrane proteins
from SMA-accessible and SMA-resistant membranes. Biochim. Biophys. Acta - Biomembr..

[19] Moad G., Rizzardo E., Thang S. H. (2009). Living
radical polymerization by
the RAFT process–a second update. Aust.
J. Chem..

[20] Dörr J. M., Scheidelaar S., Koorengevel M. C., Dominguez J. J., Schäfer M., van Walree C. A., Killian J. A. (2016). The styrene–maleic
acid copolymer: a versatile tool in membrane research. Eur. Biophys. J..

[21] Moad, G. ; Solomon, D. H. The chemistry of radical polymerization; Elsevier: 2005.

[22] Matyjaszewski, K. ; Davis, T. P. Handbook of radical polymerization; Wiley Online Library: 2002; Vol. 922.

[23] Lessard B., Maric M. (2010). One-step poly (styrene-alt-maleic anhydride)-block-poly (styrene)
copolymers with highly alternating styrene/maleic anhydride sequences
are possible by nitroxide-mediated polymerization. Macromolecules.

[24] Klumperman B. (2010). Mechanistic
considerations on styrene–maleic anhydride copolymerization
reactions. Polym. Chem..

[25] Klumperman B., Heuts J. P. (2020). The solution copolymerization
of styrene and maleic
anhydride in a continuous stirred tank reactor and its theoretical
modelling. Polymer.

[26] Hill D. J., O’Donnell J. H., O’Sullivan P.
W. (1985). Analysis of the mechanism
of copolymerization of styrene and maleic anhydride. Macromolecules.

[27] Montaudo M. S. (2001). Determination
of the compositional distribution and compositional drift in styrene/maleic
anhydride copolymers. Macromolecules.

[28] Jamshad M., Grimard V., Idini I., Knowles T. J., Dowle M. R., Schofield N., Sridhar P., Lin Y., Finka R., Wheatley M. (2015). Structural analysis of a nanoparticle containing
a lipid bilayer used for detergent-free extraction of membrane proteins. Nano Res..

[29] Corrigan N., Jung K., Moad G., Hawker C. J., Matyjaszewski K., Boyer C. (2020). Reversible-deactivation radical polymerization
(Controlled/living
radical polymerization): From discovery to materials design and applications. Prog. Polym. Sci..

[30] Smith A. A., Autzen H. E., Laursen T., Wu V., Yen M., Hall A., Hansen S. D., Cheng Y., Xu T. (2017). Controlling
styrene maleic acid lipid particles through RAFT. Biomacromolecules.

[31] Willcock H., O’Reilly R. K. (2010). End group
removal and modification of RAFT polymers. Polym.
Chem..

[32] Keddie D. J., Moad G., Rizzardo E., Thang S. H. (2012). RAFT agent design
and synthesis. Macromolecules.

[33] Perrier S. (2017). 50th Anniversary
Perspective: RAFT Polymerization— A User Guide. Macromolecules.

[34] Hall S. C., Tognoloni C., Price G. J., Klumperman B., Edler K. J., Dafforn T. R., Arnold T. (2018). Influence of poly (styrene-co-maleic
acid) copolymer structure on the properties and self-assembly of SMALP
nanodiscs. Biomacromolecules.

[35] Neville G. M., Morrison K. A., Shilliday E. R., Doutch J., Dalgliesh R., Price G. J., Edler K. J. (2023). The effect
of polymer end-group on
the formation of styrene–maleic acid lipid particles (SMALPs). Soft Matter.

[36] Xue M., Cheng L., Faustino I., Guo W., Marrink S. J. (2018). Molecular
mechanism of lipid nanodisk formation by styrene-maleic acid copolymers. Biophys. J..

[37] Ravula T., Hardin N. Z., Bai J., Im S.-C., Waskell L., Ramamoorthy A. (2018). Effect of polymer charge on functional
reconstitution
of membrane proteins in polymer nanodiscs. Chem.
Commun..

[38] Hawkins O. P., Jahromi C. P. T., Gulamhussein A. A., Nestorow S., Bahra T., Shelton C., Owusu-Mensah Q. K., Mohiddin N., O’rourke H., Ajmal M. (2021). Membrane protein extraction and purification using partially-esterified
SMA polymers. Biochim. Biophys. Acta - Biomembr..

[39] Korotych O., Mondal J., Gattás-Asfura K. M., Hendricks J., Bruce B. D. (2019). Evaluation of commercially available
styrene-co-maleic
acid polymers for the extraction of membrane proteins from spinach
chloroplast thylakoids. Eur. Polym. J..

[40] Burridge K. M., Harding B. D., Sahu I. D., Kearns M. M., Stowe R. B., Dolan M. T., Edelmann R. E., Dabney-Smith C., Page R. C., Konkolewicz D., Lorigan G. A. (2020). Simple derivatization
of RAFT-synthesized styrene–maleic anhydride copolymers for
lipid disk formulations. Biomacromolecules.

[41] Esmaili M., Acevedo-Morantes C., Wille H., Overduin M. (2020). The effect of hydrophobic
alkyl sidechains on size and solution behaviors of nanodiscs formed
by alternating styrene maleamic copolymer. Biochim.
Biophys. Acta - Biomembr..

[42] Hall S. C. L., Tognoloni C., Charlton J., Bragginton E. C., Rothnie A. J., Sridhar P., Wheatley M., Knowles T. J., Arnold T., Edler K. J., Dafforn T. R. (2018). An acid-compatible
co-polymer for the solubilization of membranes and proteins into lipid
bilayer-containing nanoparticles. Nanoscale.

[43] Beriashvili D., Spencer N. R., Dieckmann T., Overduin M., Palmer M. (2020). Characterization
of multimeric daptomycin bound to lipid nanodiscs formed by calcium-tolerant
styrene-maleic acid copolymer. Biochim. Biophys.
Acta - Biomembr..

[44] Ravula T., Hardin N. Z., Ramadugu S. K., Cox S. J., Ramamoorthy A. (2018). Formation
of pH-Resistant Monodispersed Polymer–Lipid Nanodiscs. Angew. Chem., Int. Ed. Engl..

[45] Ravula T., Hardin N. Z., Ramadugu S. K., Ramamoorthy A. (2017). pH tunable
and divalent metal ion tolerant polymer lipid nanodiscs. Langmuir.

[46] Lindhoud S., Carvalho V., Pronk J. W., Aubin-Tam M.-E. (2016). SMA-SH:
modified styrene–maleic acid copolymer for functionalization
of lipid nanodiscs. Biomacromolecules.

[47] Fiori M. C., Jiang Y., Altenberg G. A., Liang H. (2017). Polymer-encased nanodiscs
with improved buffer compatibility. Sci. Rep..

[48] Glueck D., Grethen A., Das M., Mmeka O. P., Patallo E. P., Meister A., Rajender R., Kins S., Räschle M., Victor J. (2022). Electroneutral
Polymer Nanodiscs Enable Interference-Free
Probing of Membrane Proteins in a Lipid-Bilayer Environment. Small.

[49] Farrelly M. D., Korneev D., Martin L. L., Thang S. H. (2025). Tethering Efficiency
of RAFT-Synthesised SMA Polymers and Associated SMALPs on Gold Surfaces. ChemPlusChem..

[50] Ball, L. E. ; Smith, M.-P. ; Motloung, B. ; Pfukwa, R. ; Klumperman, B. , Aqueous solution behavior of poly­(styrene-*alt*-maleic acid)-*b*-poly­(*N*-acryloylmorpholine) double hydrophilic block copolymers in the absence and presence of divalent cations and phospholipids. Macromolecules **(Under Review)**. 10.1021/acs.macromol.5c01099 PMC12257593 41769215

[51] Ravula T., Ramadugu S. K., Di Mauro G., Ramamoorthy A. (2017). Bioinspired,
Size-Tunable Self-Assembly of Polymer–Lipid Bilayer Nanodiscs. Angew. Chem., Int. Ed. Engl..

[52] Sauvage E., Amos D., Antalek B., Schroeder K., Tan J., Plucktaveesak N., Colby R. H. (2004). Amphiphilic maleic
acid-containing alternating copolymers—1. Dissociation behavior
and compositions. J. Polym. Sci., Part B: Polym.
Phys..

[53] Oluwole A. O., Danielczak B., Meister A., Babalola J. O., Vargas C., Keller S. (2017). Solubilization of membrane proteins into functional
lipid-bilayer nanodiscs using a diisobutylene/maleic acid copolymer. Angew. Chem., Int. Ed. Engl..

[54] Gulamhussein A. A., Uddin R., Tighe B. J., Poyner D. R., Rothnie A. J. (2020). A comparison
of SMA (styrene maleic acid) and DIBMA (di-isobutylene maleic acid)
for membrane protein purification. Biochim.
Biophys. Acta - Biomembr..

[55] Le
Bon C., Michon B., Popot J.-L., Zoonens M. (2021). Amphipathic environments
for determining the structure of membrane proteins by single-particle
electron cryo-microscopy. Q. Rev. Biophys..

[56] Marconnet A., Michon B., Le Bon C., Giusti F., Tribet C., Zoonens M. (2020). Solubilization and
stabilization of membrane proteins
by cycloalkane-modified amphiphilic polymers. Biomacromolecules.

[57] Higgins A. J., Flynn A. J., Marconnet A., Musgrove L. J., Postis V. L. G., Lippiat J. D., Chung C.-w., Ceska T., Zoonens M., Sobott F., Muench S. P. (2021). Cycloalkane-modified
amphiphilic
polymers provide direct extraction of membrane proteins for CryoEM
analysis. Commun. Biol..

[58] Yasuhara K., Arakida J., Ravula T., Ramadugu S. K., Sahoo B., Kikuchi J.-i., Ramamoorthy A. (2017). Spontaneous
lipid nanodisc fomation
by amphiphilic polymethacrylate copolymers. J. Am. Chem. Soc..

[59] Overduin M., Esmaili M. (2019). Memtein: The fundamental
unit of membrane-protein structure
and function. Chem. Phys. Lipids.

[60] Mitrofanova G. (2002). Complexation
of calcium ions with dicarboxylic acids in aqueous solutions. Russ. J. Appl. Chem..

[61] Oluwole A. O., Klingler J., Danielczak B., Babalola J. O., Vargas C., Pabst G., Keller S. (2017). Formation of Lipid-Bilayer Nanodiscs
by Diisobutylene/Maleic Acid (DIBMA) Copolymer. Langmuir.

[62] Cuevas
Arenas R., Klingler J., Vargas C., Keller S. (2016). Influence
of lipid bilayer properties on nanodisc formation mediated by styrene/maleic
acid copolymers. Nanoscale.

[63] Danielczak B., Rasche M., Lenz J., Perez Patallo E., Weyrauch S., Mahler F., Agbadaola M. T., Meister A., Babalola J. O., Vargas C. (2022). A bioinspired
glycopolymer for capturing membrane proteins in native-like lipid-bilayer
nanodiscs. Nanoscale.

[64] Tribet C., Audebert R., Popot J.-L. (1996). Amphipols:
polymers that keep membrane
proteins soluble in aqueous solutions. Proc.
Natl. Acad. Sci. U. S. A..

[65] Lavington S., Watts A. (2021). Detergent-free solubilisation
& purification of a G protein coupled
receptor using a polymethacrylate polymer. Biochim.
Biophys. Acta - Biomembr..

[66] Smith A. A., Autzen H. E., Faust B., Mann J. L., Muir B. W., Howard S., Postma A., Spakowitz A. J., Cheng Y., Appel E. A. (2020). Lipid nanodiscs via ordered copolymers. Chem..

[67] Timcenko M., Autzen A. A., Autzen H. E. (2022). Characterization
of divalent cation
interactions with AASTY nanodiscs. ACS Appl.
Polym. Mater..

[68] Kopf A. H., Lijding O., Elenbaas B. O., Koorengevel M. C., Dobruchowska J. M., van Walree C. A., Killian J. A. (2022). Synthesis and evaluation
of a library of alternating amphipathic copolymers to solubilize and
study membrane proteins. Biomacromolecules.

[69] Baruah S. D., Laskar N. C. (1996). Styrene-maleic anhydride copolymers: Synthesis, characterization,
and thermal properties. J. Appl. Polym. Sci..

[70] Chernikova E. V., Zaitsev S. D., Plutalova A. V., Mineeva K. O., Zotova O. S., Vishnevetsky D. V. (2018). Control
over the relative reactivities of monomers
in RAFT copolymerization of styrene and acrylic acid. RSC Adv..

[71] Chernikova E., Zaitsev S., Plutalova A., Mineeva K., Zotova O., Vishnevetsky D. (2018). Control over
the relative reactivities of monomers
in RAFT copolymerization of styrene and acrylic acid. RSC Adv..

[72] Williams, J. J. , Formulation of carpet cleaners. In Handbook for cleaning/decontamination of surfaces; Elsevier: 2007; pp 103–123.

[73] Chakraborty S., N. L. M. (2020). A carbon nanotube reinforced functionalized styrene–maleic
anhydride copolymer as an advanced electrode material for efficient
energy storage applications. New J. Chem..

[74] Grime R. L., Logan R. T., Nestorow S. A., Sridhar P., Edwards P. C., Tate C. G., Klumperman B., Dafforn T. R., Poyner D. R., Reeves P. J., Wheatley M. (2021). Differences
in SMA-like polymer architecture
dictate the conformational changes exhibited by the membrane protein
rhodopsin encapsulated in lipid nano-particles. Nanoscale.

[75] Zhou R., Zhang S., Nguyen H. T., Ding H., Gaffney A., Kappes J. C., Smith III A. B., Sodroski J. G. (2023). Conformations of
human immunodeficiency virus envelope glycoproteins in detergents
and styrene-maleic acid lipid particles. J.
Virol..

[76] Buachi C., Thammachai C., Tighe B. J., Topham P. D., Molloy R., Punyamoonwongsa P. (2023). Encapsulation of propolis extracts in aqueous formulations
by using nanovesicles of lipid and poly (styrene-alt-maleic acid). Artif. Cells Nanomed. Biotechnol..

[77] Sforcin J. M. (2016). Biological
properties and therapeutic applications of propolis. Phytother. Res..

[78] Torgersen M. L., Judge P. J., Bada
Juarez J. F., Pandya A. D., Fusser M., Davies C. W., Maciejewska M. K., Yin D. J., Maelandsmo G. M., Skotland T. (2020). Physicochemical characterization, toxicity
and in vivo biodistribution studies of a discoidal, lipid-based drug
delivery vehicle: Lipodisq nanoparticles containing doxorubicin. J. Biomed. Nanotechnol..

[79] Marrink S. J., Corradi V., Souza P. C., Ingolfsson H. I., Tieleman D. P., Sansom M. S. (2019). Computational modeling
of realistic
cell membranes. Chem. Rev..

[80] Jefferies D., Khalid S. (2021). Atomistic and coarse-grained
simulations of membrane
proteins: a practical guide. Methods.

[81] Marrink S. J., Monticelli L., Melo M. N., Alessandri R., Tieleman D. P., Souza P. C. (2023). Two decades
of Martini: Better beads,
broader scope. Wiley Interdiscip. Rev. Comput.
Mol. Sci..

[82] Vattulainen I., Róg T. (2016). Lipid membranes: Theory and simulations bridged to
experiments. Biochim. Biophys. Acta - Biomembr..

[83] Schmidt V., Sidore M., Bechara C., Duneau J.-P., Sturgis J. N. (2019). The lipid
environment of Escherichia coli Aquaporin Z. Biochim. Biophys. Acta - Biomembr..

[84] Colbasevici A., Voskoboynikova N., Orekhov P. S., Bozdaganyan M. E., Karlova M. G., Sokolova O. S., Klare J. P., Mulkidjanian A. Y., Shaitan K. V., Steinhoff H.-J. (2020). Lipid dynamics
in nanoparticles formed
by maleic acid-containing copolymers: EPR spectroscopy and molecular
dynamics simulations. Biochim. Biophys. Acta
- Biomembr..

[85] Jeong C., Franklin R., Edler K. J., Vanommeslaeghe K., Krueger S., Curtis J. E. (2022). Styrene–Maleic Acid Copolymer
Nanodiscs to Determine the Shape of Membrane Proteins. J. Phys. Chem. B.

[86] Orekhov P. S., Bozdaganyan M. E., Voskoboynikova N., Mulkidjanian A. Y., Karlova M. G., Yudenko A., Remeeva A., Ryzhykau Y. L., Gushchin I., Gordeliy V. I. (2022). Mechanisms of formation,
structure, and dynamics of lipoprotein discs stabilized by amphiphilic
copolymers: A comprehensive review. Nanomater..

